# Supplementary material for: Nitropyridine-1-Oxides as Excellent π-Hole Donors: Interplay between σ-Hole (Halogen, Hydrogen, Triel, and Coordination Bonds) and π-Hole Interactions
Source: Int J Mol Sci. 2019 Jul 12;20(14):3440. doi: 10.3390/ijms20143440 (PMC6678756; doi:10.3390/ijms20143440)
Supplement: Supplementary file 1 [file ijms-20-03440-s001.pdf]

Supplementary Materials

Binary complexes

*Complex **2** (**1** + HF)*

C	-0.5028736	0.0009953	-1.1764833
C	0.8675120	-0.0005488	-1.1989648
C	1.5578617	-0.0009572	0.0000000
C	0.8675120	-0.0005488	1.1989648
C	-0.5028736	0.0009953	1.1764833
H	-1.1325369	0.0018979	-2.0537283
H	1.3998690	-0.0011158	-2.1400847
H	1.3998690	-0.0011158	2.1400847
H	-1.1325369	0.0018979	2.0537283
N	3.0160222	-0.0011878	0.0000000
O	3.5700810	-0.0009432	1.0776226
O	3.5700810	-0.0009432	-1.0776226
N	-1.1903362	0.0024557	0.0000000
O	-2.4458043	0.0059119	0.0000000
F	-5.1355612	-0.0041490	0.0000000
H	-4.2062854	-0.0026445	0.0000000

*Complex **3** (**1** + CF_3I)*

C	0.7918062	0.9209277	-1.1759269
C	1.8722905	0.0781121	-1.1992410
C	2.4181174	-0.3443498	0.0000000
C	1.8722905	0.0781121	1.1992410
C	0.7918062	0.9209277	1.1759269
H	0.2933833	1.3054201	-2.0532475
H	2.2901278	-0.2519853	-2.1400975
H	2.2901278	-0.2519853	2.1400975
H	0.2933833	1.3054201	2.0532475

N	3.5700949	-1.2374191	0.0000000
O	4.0087441	-1.5769504	1.0775311
O	4.0087441	-1.5769504	-1.0775311
N	0.2543257	1.3496521	0.0000000
O	-0.7363862	2.1303500	0.0000000
C	-4.9210386	-0.6581893	0.0000000
I	-3.2051869	0.6401419	0.0000000
F	-4.5484478	-1.9338226	0.0000000
F	-5.6720910	-0.4487058	-1.0752005
F	-5.6720910	-0.4487058	1.0752005

*Complex **4** (**1** + AgCl)*

C	-0.4234634	0.4644945	-1.1748883
C	0.8883977	0.0613255	-1.2008535
C	1.5439386	-0.1359325	0.0000000
C	0.8883977	0.0613255	1.2008535
C	-0.4234634	0.4644945	1.1748883
H	-1.0272952	0.6484466	-2.0514958
H	1.3977778	-0.0968924	-2.1412353
H	1.3977778	-0.0968924	2.1412353
H	-1.0272952	0.6484466	2.0514958
N	2.9486907	-0.5542312	0.0000000
O	3.4748676	-0.7092863	1.0780925
O	3.4748676	-0.7092863	-1.0780925
N	-1.0637732	0.6701268	0.0000000
O	-2.2722228	1.1048197	0.0000000
Ag	-3.9997437	-0.2018644	0.0000000
Cl	-5.7774587	-1.6190944	0.0000000

*Complex **5** (**1** + BF_3)*

C	-0.2023831	-0.4636177	-1.1764893
C	1.1130565	-0.0630370	-1.2049806

C	1.7589733	0.1309796	0.0000000
C	1.1130565	-0.0630370	1.2049806
C	-0.2023831	-0.4636177	1.1764893
H	-0.8224670	-0.6266614	-2.0451705
H	1.6232825	0.1028354	-2.1430095
H	1.6232825	0.1028354	2.1430095
H	-0.8224670	-0.6266614	2.0451705
N	3.1686133	0.5641039	0.0000000
O	3.6896428	0.7253422	1.0778231
O	3.6896428	0.7253422	-1.0778231
N	-0.8178152	-0.6648915	0.0000000
O	-2.0715733	-1.0602788	0.0000000
F	-4.2935195	-0.3520762	0.0000000
F	-2.7407494	0.9120670	-1.1387438
F	-2.7407494	0.9120670	1.1387438
B	-3.0654431	0.2083058	0.0000000

Complex ***6*** *(**1** + NO_3^-)*

H	-0.1388931	-0.9040769	2.1325525
C	0.3332524	-0.6546016	1.1938588
C	1.4994984	0.0611649	-1.1692609
C	1.4994984	0.0611649	1.1692609
C	-0.2482194	-1.0347390	0.0000000
C	0.3332524	-0.6546016	-1.1938588
N	2.1044614	0.4035791	0.0000000
H	2.0182093	0.4142906	2.0471910
H	-0.1388931	-0.9040769	-2.1325525
H	2.0182093	0.4142906	-2.0471910
N	-1.4465153	-1.8462765	0.0000000
O	-1.9003805	-2.1833774	1.0752511
O	-1.9003805	-2.1833774	-1.0752511
O	-0.5673015	2.1045027	0.0000000

N	-1.8114555	2.0011552	0.0000000
O	-2.3360915	0.8659499	0.0000000
O	-2.5217169	3.0124784	0.0000000
O	3.2034656	1.0265508	0.0000000

*Complex **7** (**1** + BF_4^-)*

H	0.1187247	-1.0693637	2.1340393
C	0.5446254	-0.7468267	1.1953234
C	1.5774523	0.1503194	-1.1712420
C	1.5774523	0.1503194	1.1712420
C	0.0378845	-1.2199550	0.0000000
C	0.5446254	-0.7468267	-1.1953234
N	2.1191965	0.5847469	0.0000000
H	2.0284814	0.5878145	2.0483918
H	0.1187247	-1.0693637	-2.1340393
H	2.0284814	0.5878145	-2.0483918
N	-1.0167606	-2.2126418	0.0000000
O	-1.4129755	-2.6141762	1.0754601
O	-1.4129755	-2.6141762	-1.0754601
F	-2.2852026	0.3971998	0.0000000
O	3.*1009777*	1.3734929	0.0000000
B	-1.9990884	1.7803082	0.0000000
F	-0.5965569	1.9499787	0.0000000
F	-2.5365335	2.3656677	1.1487086
F	-2.5365335	2.3656677	-1.1487086

*Complex **8** (**1** + Me_3N)*

H	3.1794118	0.0008216	2.0539596
C	2.6378696	-0.3159979	1.1752675
C	1.4566763	-1.0090147	-1.1966174
C	1.4566763	-1.0090147	1.1966174
N	3.2364214	0.0415215	0.0000000

C	2.6378696	-0.3159979	-1.1752675
C	0.8537748	-1.3545550	0.0000000
H	0.9965060	-1.2757117	2.1375925
H	3.1794118	0.0008216	-2.0539596
H	0.9965060	-1.2757117	-2.1375925
N	-0.4014012	-2.0814484	0.0000000
O	-0.8813911	-2.3697284	1.0764834
O	-0.8813911	-2.3697284	-1.0764834
N	-1.4750243	0.6166392	0.0000000
C	-1.1821185	1.3650036	1.1964583
H	-1.8071556	2.2697322	1.3022285
H	-0.1350086	1.6789540	1.1934407
H	-1.3476397	0.7358272	2.0742887
C	-2.8221133	0.0963544	0.0000000
H	-3.5864853	0.8939576	0.0000000
H	-2.9756916	-0.5283015	0.8821160
H	-2.9756916	-0.5283015	-0.8821160
C	-1.1821185	1.3650036	-1.1964583
H	-1.8071556	2.2697322	-1.3022285
H	-1.3476397	0.7358272	-2.0742887
H	-0.1350086	1.6789540	-1.1934407
O	4.3119109	0.6843620	0.0000000

*Complex **9** (**1** + $MeNO_2$)*

H	0.4930678	-1.5428938	2.1376230
C	0.9077628	-1.2083562	1.1971233
C	1.9629643	-0.3368284	-1.1751715
C	1.9629643	-0.3368284	1.1751715
C	0.3714590	-1.6481061	0.0000000
C	0.9077628	-1.2083562	-1.1971233
N	2.4963476	0.1100394	0.0000000
H	2.4443885	0.0667905	2.0530593

H	0.4930678	-1.5428938	-2.1376230
H	2.4443885	0.0667905	-2.0530593
N	-0.7647099	-2.5493741	0.0000000
O	-1.2019621	-2.8986347	1.0769150
O	-1.2019621	-2.8986347	-1.0769150
O	-0.3934324	1.7368968	0.0000000
N	-1.5808564	1.5032949	0.0000000
O	-2.0832185	0.4029589	0.0000000
O	3.4567046	0.9136011	0.0000000
C	-2.5131776	2.6591993	0.0000000
H	-1.9308172	3.5744536	0.0000000
H	-3.1353710	2.5684407	0.8880289
H	-3.1353710	2.5684407	-0.8880289

*Complex **10** (**1** + CO)*

C	0.2805769	-0.6695251	0.0000000
N	-2.1690335	0.6022333	0.0000000
C	-0.3357445	-0.3498812	-1.1979590
C	-0.3357445	-0.3498812	1.1979590
C	-1.5509137	0.2806361	1.1767302
C	-1.5509137	0.2806361	-1.1767302
H	0.1381053	-0.5921149	-2.1389816
H	0.1381053	-0.5921149	2.1389816
H	-2.1078449	0.5708904	2.0549798
H	-2.1078449	0.5708904	-2.0549798
O	-3.2776248	1.1804940	0.0000000
N	1.5740332	-1.3251740	0.0000000
O	2.0744449	-1.5744702	-1.0769978
O	2.0744449	-1.5744702	1.0769978
C	3.1103510	1.4604094	0.0000000
O	4.0456029	2.0814422	0.0000000

Ternary complexes

*Complex **11** (**1** + NO_3^- + HF)*

H	-0.6185220	-0.8683427	2.1338376
C	-0.1473873	-0.6156528	1.1959681
C	1.0220438	0.0987903	-1.1670797
C	1.0220438	0.0987903	1.1670797
C	-0.7289426	-0.9829307	0.0000000
C	-0.1473873	-0.6156528	-1.1959681
N	1.6098968	0.4332201	0.0000000
H	1.5505927	0.4424778	2.0428967
H	-0.6185220	-0.8683427	-2.1338376
H	1.5505927	0.4424778	-2.0428967
N	-1.9345664	-1.8005337	0.0000000
O	-2.3837248	-2.1345002	1.0752658
O	-2.3837248	-2.1345002	-1.0752658
O	-1.0021241	2.1079454	0.0000000
N	-2.2442624	1.9872101	0.0000000
O	-2.7505314	0.8411967	0.0000000
O	-2.9703949	2.9827862	0.0000000
O	2.7290106	1.0782338	0.0000000
F	4.5668479	-0.5885591	0.0000000
H	3.8790620	0.0958863	0.0000000

*Complex **12** (**1** + BF_4^- + HF)*

H	-0.3488855	-1.0853902	2.1347285
C	0.0775070	-0.7616203	1.1971260
C	1.1160774	0.1326940	-1.1690266
C	1.1160774	0.1326940	1.1690266
C	-0.4285813	-1.2232797	0.0000000
C	0.0775070	-0.7616203	-1.1971260
N	1.6432803	0.5548437	0.0000000
H	1.5785848	0.5623452	2.0441220

H	-0.3488855	-1.0853902	-2.1347285
H	1.5785848	0.5623452	-2.0441220
N	-1.4962967	-2.2173639	0.0000000
O	-1.8896919	-2.6125518	1.0753175
O	-1.8896919	-2.6125518	-1.0753175
F	-2.7041682	0.3263054	0.0000000
O	2.6439285	1.3633260	0.0000000
B	-2.4292471	1.7149332	0.0000000
F	-1.0253938	1.8893958	0.0000000
F	-2.9671169	2.2923790	1.1488888
F	-2.9671169	2.2923790	-1.1488888
F	4.7173199	-0.0228385	0.0000000
H	3.9462084	0.5589665	0.0000000

*Complex **13** (**1** + Me_3N + HF)*

H	2.7607297	0.0723081	2.0494275
C	2.2140862	-0.2419669	1.1728376
C	1.0351490	-0.9428393	-1.1983674
C	1.0351490	-0.9428393	1.1983674
N	2.7923219	0.1162268	0.0000000
C	2.2140862	-0.2419669	-1.1728376
C	0.4361201	-1.2824889	0.0000000
H	0.5792649	-1.2182307	2.1385784
H	2.7607297	0.0723081	-2.0494275
H	0.5792649	-1.2182307	-2.1385784
N	-0.8173091	-2.0291258	0.0000000
O	-1.2886121	-2.3223561	1.0766028
O	-1.2886121	-2.3223561	-1.0766028
N	-1.8470719	0.6482186	0.0000000
C	-1.5543401	1.3964474	1.1971305
H	-2.1793000	2.3003669	1.3021978
H	-0.5075900	1.7117583	1.1942837

H	-1.7205651	0.7673968	2.0749073
C	-3.1941707	0.1260599	0.0000000
H	-3.9580102	0.9230471	0.0000000
H	-3.3475480	-0.4980740	0.8824950
H	-3.3475480	-0.4980740	-0.8824950
C	-1.5543401	1.3964474	-1.1971305
H	-2.1793000	2.3003669	-1.3021978
H	-1.7205651	0.7673968	-2.0749073
H	-0.5075900	1.7117583	-1.1942837
O	3.8799030	0.7851224	0.0000000
F	5.6413098	-1.0533185	0.0000000
H	5.0843583	-0.2833625	0.0000000

*Complex **14** (**1** + $MeNO_2$ + HF)*

H	0.0389615	-1.5274684	2.1383045
C	0.4493809	-1.1863682	1.1986399
C	1.4978762	-0.3030095	-1.1726907
C	1.4978762	-0.3030095	1.1726907
C	-0.0795361	-1.6236698	0.0000000
C	0.4493809	-1.1863682	-1.1986399
N	2.0107659	0.1413401	0.0000000
H	1.9824019	0.1016815	2.0485377
H	0.0389615	-1.5274684	-2.1383045
H	1.9824019	0.1016815	-2.0485377
N	-1.2187066	-2.5357698	0.0000000
O	-1.6508761	-2.8824777	1.0769259
O	-1.6508761	-2.8824777	-1.0769259
O	-0.8109728	1.7340988	0.0000000
N	-1.9998565	1.5061918	0.0000000
O	-2.5049562	0.4068648	0.0000000
O	2.9760165	0.9751728	0.0000000
C	-2.9270540	2.6655385	0.0000000

H	-2.3405538	3.5781069	0.0000000
H	-3.5495935	2.5771859	0.8880549
H	-3.5495935	2.5771859	-0.8880549
F	5.0218862	-0.5331494	0.0000000
H	4.3366656	0.1261879	0.0000000

*Complex **15** (**1** + CO + HF)*

C	0.7910419	-0.6499092	0.0000000
N	-1.6111215	0.6652808	0.0000000
C	0.1864418	-0.3232067	-1.1994442
C	0.1864418	-0.3232067	1.1994442
C	-1.0172041	0.3341236	1.1742536
C	-1.0172041	0.3341236	-1.1742536
H	0.6537211	-0.5794986	-2.1397538
H	0.6537211	-0.5794986	2.1397538
H	-1.5742235	0.6305107	2.0505442
H	-1.5742235	0.6305107	-2.0505442
O	-2.7250468	1.2814188	0.0000000
N	2.0792905	-1.3353499	0.0000000
O	2.5695946	-1.5919609	-1.0771078
O	2.5695946	-1.5919609	1.0771078
C	3.5870779	1.4884236	0.0000000
O	4.4926299	2.1513676	0.0000000
H	-3.8809838	0.1355192	0.0000000
F	-4.3695479	-0.6766873	0.0000000

*Complex **16** (**1** + NO_3^- + CF_3I)*

C	0.0970814	0.4897038	-1.1681831
C	1.0609526	-0.4838243	-1.1960616
C	1.5372769	-0.9806989	0.0000000
C	1.0609526	-0.4838243	1.1960616
C	0.0970814	0.4897038	1.1681831
H	-0.3316846	0.9509110	-2.0445908

H	1.4571843	-0.8432666	-2.1338711
H	1.4571843	-0.8432666	2.1338711
H	-0.3316846	0.9509110	2.0445908
N	2.5091343	-2.0644928	0.0000000
O	2.8643490	-2.4972689	1.0753282
O	2.8643490	-2.4972689	-1.0753282
N	-0.3941823	0.9583279	0.0000000
O	-1.3293174	1.8428482	0.0000000
C	-5.6179602	-0.2845185	0.0000000
I	-3.7071417	0.7197752	0.0000000
F	-5.4794881	-1.6144381	0.0000000
F	-6.3469017	0.0345937	-1.0753048
F	-6.3469017	0.0345937	1.0753048
N	3.7240211	1.5371804	0.0000000
O	3.9323962	0.3016160	0.0000000
O	4.6733683	2.3226385	0.0000000
O	2.5499308	1.9600646	0.0000000

*Complex **17** (**1** + BF_4^- + CF_3I)*

C	-0.0403460	0.3936953	-1.1700725
C	0.8623928	-0.6368541	-1.1971160
C	1.2990981	-1.1660676	0.0000000
C	0.8623928	-0.6368541	1.1971160
C	-0.0403460	0.3936953	1.1700725
H	-0.4346178	0.8855597	-2.0457745
H	1.2387243	-1.0175717	-2.1348443
H	1.2387243	-1.0175717	2.1348443
H	-0.4346178	0.8855597	2.0457745
N	2.2182770	-2.2967047	0.0000000
O	2.5544706	-2.7425926	1.0754899
O	2.5544706	-2.7425926	-1.0754899
N	-0.5039175	0.8894840	0.0000000

O	-1.3821637	1.8217603	0.0000000
C	-5.7755331	-0.1598398	0.0000000
I	-3.8405681	0.7903887	0.0000000
F	-5.6691854	-1.4915652	0.0000000
F	-6.4930966	0.1792780	-1.0751681
F	-6.4930966	0.1792780	1.0751681
F	3.7609966	0.0954525	0.0000000
F	2.2988928	1.8619631	0.0000000
F	4.2754624	2.0121980	1.1489826
F	4.2754624	2.0121980	-1.1489826
B	3.6681236	1.5077032	0.0000000

*Complex **18** (**1** + Me_3N + CF_3I)*

C	1.3193702	0.3116167	1.1742338
C	0.2972737	-0.6015762	1.1978906
C	-0.2279249	-1.0499866	0.0000000
C	0.2972737	-0.6015762	-1.1978906
C	1.3193702	0.3116167	-1.1742338
H	1.7922404	0.7267043	2.0517452
H	-0.0984407	-0.9585344	2.1381381
H	-0.0984407	-0.9585344	-2.1381381
H	1.7922404	0.7267043	-2.0517452
N	-1.3247719	-2.0065749	0.0000000
O	-1.7390840	-2.3787623	-1.0764689
O	-1.7390840	-2.3787623	1.0764689
N	1.8189384	0.7829847	0.0000000
O	2.7463050	1.6453034	0.0000000
C	7.0751428	-0.8111987	0.0000000
I	5.2644601	0.3498032	0.0000000
F	6.8055910	-2.1135413	0.0000000
F	7.8096800	-0.5443872	1.0750483
F	7.8096800	-0.5443872	-1.0750483

N	-2.8124334	0.4735301	0.0000000
C	-4.0483262	-0.2742216	0.0000000
H	-4.0908561	-0.9156589	-0.8826674
H	-4.0908561	-0.9156589	0.8826674
H	-4.9394779	0.3775266	0.0000000
C	-2.6545461	1.2619267	1.1968808
H	-2.7079658	0.6138255	2.0750403
H	-1.6786344	1.7547555	1.1935194
H	-3.4277888	2.0432780	1.3025730
C	-2.6545461	1.2619267	-1.1968808
H	-1.6786344	1.7547555	-1.1935194
H	-2.7079658	0.6138255	-2.0750403
H	-3.4277888	2.0432780	-1.3025730

Complex ***19*** *(**1** + $MeNO_2$ + CF_3I)*

C	-0.4884431	0.0630220	-1.1739602
C	0.4193436	-0.9630454	-1.1982356
C	0.8800488	-1.4753204	0.0000000
C	0.4193436	-0.9630454	1.1982356
C	-0.4884431	0.0630220	1.1739602
H	-0.9052615	0.5350939	-2.0508036
H	0.7750958	-1.3606046	-2.1381712
H	0.7750958	-1.3606046	2.1381712
H	-0.9052615	0.5350939	2.0508036
N	1.8710202	-2.5417045	0.0000000
O	2.2490408	-2.9496520	1.0769562
O	2.2490408	-2.9496520	-1.0769562
N	-0.9338298	0.5839902	0.0000000
O	-1.7645800	1.5383910	0.0000000
C	-6.2446590	-0.6333349	0.0000000
I	-4.3734120	0.4278465	0.0000000
F	-6.0455171	-1.9484456	0.0000000

F	-6.9645024	-0.3281123	-1.0748939
F	-6.9645024	-0.3281123	1.0748939
N	3.1949423	1.4145962	0.0000000
O	3.5485025	0.2579806	0.0000000
O	2.0473024	1.8001698	0.0000000
C	4.2473168	2.4619345	0.0000000
H	4.0920561	3.0712761	-0.8879535
H	4.0920561	3.0712761	0.8879535
H	5.2182064	1.9779414	0.0000000

*Complex **20** (**1** + CO + CF_3I)*

C	0.1366336	0.9711510	-1.1756646
C	1.1704631	0.0717916	-1.1989682
C	1.6897567	-0.3829037	0.0000000
C	1.1704631	0.0717916	1.1989682
C	0.1366336	0.9711510	1.1756646
H	-0.3408839	1.3813006	-2.0529432
H	1.5713139	-0.2790796	-2.1395496
H	1.5713139	-0.2790796	2.1395496
H	-0.3408839	1.3813006	2.0529432
N	2.7833999	-1.3424018	0.0000000
O	3.2035642	-1.7067804	1.0768930
O	3.2035642	-1.7067804	-1.0768930
N	-0.3818536	1.4225967	0.0000000
O	-1.3452432	2.2375866	0.0000000
C	-5.2526445	-0.9597884	0.0000000
I	-3.7312695	0.5619456	0.0000000
F	-4.7042433	-2.1703529	0.0000000
F	-6.0249840	-0.8576041	-1.0751448
F	-6.0249840	-0.8576041	1.0751448
C	5.1957015	0.6881381	0.0000000
O	6.3141822	0.7836216	0.0000000

*Complex **21** (**1** + NO_3^- + AgCl)*

H	-0.7276686	-0.8883012	2.1349113
C	-0.2685478	-0.6135933	1.1972655
C	0.8657324	0.1556025	-1.1673576
C	0.8657324	0.1556025	1.1673576
C	-0.8333405	-1.0032052	0.0000000
C	-0.2685478	-0.6135933	-1.1972655
N	1.4326350	0.5108981	0.0000000
H	1.3761829	0.5259532	2.0433577
H	-0.7276686	-0.8883012	-2.1349113
H	1.3761829	0.5259532	-2.0433577
N	-1.9958643	-1.8867503	0.0000000
O	-2.4243190	-2.2416577	1.0756799
O	-2.4243190	-2.2416577	-1.0756799
O	-1.2106354	2.0525049	0.0000000
N	-2.4432259	1.8592431	0.0000000
O	-2.8793023	0.6824191	0.0000000
O	2.5068267	1.2548284	0.0000000
Ag	4.4485371	0.3727351	0.0000000
Cl	6.5596412	-0.5238708	0.0000000
O	-3.2280313	2.8051907	0.0000000

*Complex **22** (**1** + BF_4^- + AgCl)*

H	0.5498154	1.0856139	2.1354919
C	0.1077523	0.7824636	1.1982255
C	-0.9694303	-0.0659091	-1.1692727
C	-0.9694303	-0.0659091	1.1692727
C	0.6321906	1.2186323	0.0000000
C	0.1077523	0.7824636	-1.1982255
N	-1.5085178	-0.4594520	0.0000000
H	-1.4474694	-0.4789004	2.0443006

H	0.5498154	1.0856139	-2.1354919
H	-1.4474694	-0.4789004	-2.0443006
N	1.7499939	2.1625923	0.0000000
O	2.1600163	2.5348573	1.0757663
O	2.1600163	2.5348573	-1.0757663
F	2.7706427	-0.4402315	0.0000000
O	-2.5221422	-1.2735781	0.0000000
B	2.4282637	-1.8157963	0.0000000
F	1.0145603	-1.9148957	0.0000000
F	2.9325296	-2.4163004	1.1492066
F	2.9325296	-2.4163004	-1.1492066
Ag	-4.5315371	-0.5487726	0.0000000
Cl	-6.6998818	0.1878516	0.0000000

*Complex **23** (**1** + Me_3N + AgCl)*

H	-2.6435816	-0.2028585	2.0495402
C	-2.1072565	0.1300016	1.1729781
C	-0.9525056	0.8706011	-1.1996271
C	-0.9525056	0.8706011	1.1996271
N	-2.6697149	-0.2374204	0.0000000
C	-2.1072565	0.1300016	-1.1729781
C	-0.3676231	1.2299310	0.0000000
H	-0.5074041	1.1635521	2.1396523
H	-2.6435816	-0.2028585	-2.0495402
H	-0.5074041	1.1635521	-2.1396523
N	0.8490228	2.0409077	0.0000000
O	1.3011104	2.3576156	1.0770848
O	1.3011104	2.3576156	-1.0770848
N	1.9360621	-0.5810807	0.0000000
C	1.6739335	-1.3397713	1.1980099
H	2.3355297	-2.2162645	1.3034350
H	0.6414783	-1.6996567	1.1948735

H	1.8142052	-0.7039460	2.0754173
C	3.2597161	-0.0001825	0.0000000
H	4.0561660	-0.7633722	0.0000000
H	3.3860069	0.6292854	0.8829388
H	3.3860069	0.6292854	-0.8829388
C	1.6739335	-1.3397713	-1.1980099
H	2.3355297	-2.2162645	-1.3034350
H	1.8142052	-0.7039460	-2.0754173
H	0.6414783	-1.6996567	-1.1948735
Ag	-5.6498077	0.0377671	0.0000000
Cl	-7.5628786	1.2665031	0.0000000
O	-3.7339752	-0.9701705	0.0000000

*Complex **24** (**1** + $MeNO_2$ + AgCl)*

H	-0.1835026	-1.5743443	2.1389788
C	0.2416147	-1.2508867	1.1995330
C	1.3241082	-0.4082573	-1.1728141
C	1.3241082	-0.4082573	1.1728141
C	-0.3017711	-1.6655870	0.0000000
C	0.2416147	-1.2508867	-1.1995330
N	1.8481464	0.0107127	0.0000000
H	1.8203775	-0.0177298	2.0488616
H	-0.1835026	-1.5743443	-2.1389788
H	1.8203775	-0.0177298	-2.0488616
N	-1.4809400	-2.5322718	0.0000000
O	-1.9245938	-2.8578479	1.0774342
O	-1.9245938	-2.8578479	-1.0774342
O	-0.8198001	1.7401237	0.0000000
N	-2.0192693	1.5698568	0.0000000
O	-2.5740892	0.4951796	0.0000000
O	2.8235328	0.8518100	0.0000000
Ag	4.8886417	0.2236199	0.0000000

Cl	7.0535671	-0.4764703	0.0000000
C	-2.8645764	2.7897914	0.0000000
H	-3.9067512	2.4889641	0.0000000
H	-2.6013493	3.3612015	-0.8879375
H	-2.6013493	3.3612015	0.8879375

*Complex **25** (**1** + CO + AgCl)*

C	0.9402820	-0.6927309	0.0000000
N	-1.3944331	0.7178363	0.0000000
C	0.3543722	-0.3398013	-1.2005278
C	0.3543722	-0.3398013	1.2005278
C	-0.8206057	0.3689759	1.1744992
C	-0.8206057	0.3689759	-1.1744992
H	0.8111145	-0.6155682	-2.1405982
H	0.8111145	-0.6155682	2.1405982
H	-1.3639211	0.6913273	2.0506773
H	-1.3639211	0.6913273	-2.0506773
O	-2.4755653	1.4162474	0.0000000
Ag	-4.3796776	0.3746114	0.0000000
Cl	-6.2657894	-0.8921076	0.0000000
N	2.2000910	-1.4376184	0.0000000
O	2.6743074	-1.7149383	-1.0777453
O	2.6743074	-1.7149383	1.0777453
C	3.5972822	1.5127086	0.0000000
O	4.4672758	2.2210623	0.0000000

*Complex **26** (**1** + NO_3^- + BF_3)*

C	0.6409283	-0.0739355	1.1709689
C	-0.5526441	0.6006394	1.2014095
C	-1.1457841	0.9326150	0.0000000
C	-0.5526441	0.6006394	-1.2014095
C	0.6409283	-0.0739355	-1.1709689

H	1.1986293	-0.3827975	2.0408599
H	-1.0302020	0.8463988	2.1372492
H	-1.0302020	0.8463988	-2.1372492
H	1.1986293	-0.3827975	-2.0408599
N	-2.3700949	1.7400322	0.0000000
O	-2.8195210	2.0647226	-1.0754842
O	-2.8195210	2.0647226	1.0754842
N	1.2124568	-0.3791162	0.0000000
O	2.3773352	-1.0284103	0.0000000
F	4.6782899	-0.8731784	0.0000000
F	3.4870209	0.7290535	1.1408601
F	3.4870209	0.7290535	-1.1408601
B	3.5817376	-0.0565011	0.0000000
N	-2.5467219	-1.9871599	0.0000000
O	-1.3080671	-2.1200490	0.0000000
O	-3.0396555	-0.8305825	0.0000000
O	-3.2879191	-2.9658127	0.0000000

Complex ***27*** *(**1** + BF_4^- + BF_3)*

C	0.7466004	0.0102516	1.1721294
C	-0.3832344	0.7901598	1.2020098
C	-0.9302684	1.1833018	0.0000000
C	-0.3832344	0.7901598	-1.2020098
C	0.7466004	0.0102516	-1.1721294
H	1.2697107	-0.3557226	2.0411813
H	-0.8416197	1.0729933	2.1372023
H	-0.8416197	1.0729933	-2.1372023
H	1.2697107	-0.3557226	-2.0411813
N	-2.1065522	2.0668182	0.0000000
O	-2.5338255	2.4146466	-1.0754818
O	-2.5338255	2.4146466	1.0754818
N	1.2861238	-0.3431520	0.0000000

O	2.3818918	-1.0948845	0.0000000
F	4.6916827	-1.1469200	0.0000000
F	3.6451228	0.5550934	1.1399473
F	3.6451228	0.5550934	-1.1399473
B	3.6771584	-0.2337588	0.0000000
F	-3.0482505	-2.5207337	1.1490177
F	-3.0482505	-2.5207337	-1.1490177
F	-2.9799059	-0.5387651	0.0000000
F	-1.1563531	-1.9272674	0.0000000
B	-2.5727845	-1.8987492	0.0000000

*Complex **28** (**1** + Me_3N + BF_3)*

C	1.8368431	0.2821542	1.1753495
C	0.6211261	0.9213752	1.2039503
C	0.0127602	1.2216452	0.0000000
C	0.6211261	0.9213752	-1.2039503
C	1.8368431	0.2821542	-1.1753495
H	2.4177735	0.0108538	2.0439159
H	0.1547226	1.1851053	2.1416465
H	0.1547226	1.1851053	-2.1416465
H	2.4177735	0.0108538	-2.0439159
N	-1.2642557	1.9486573	0.0000000
O	-1.7340217	2.2347698	-1.0769344
O	-1.7340217	2.2347698	1.0769344
N	2.4022629	-0.0346216	0.0000000
O	3.5635812	-0.6627994	0.0000000
F	5.8719231	-0.3927233	0.0000000
F	4.5913705	1.1442666	1.1404401
F	4.5913705	1.1442666	-1.1404401
B	4.7665025	0.3864117	0.0000000
N	-2.1276720	-0.7191018	0.0000000
C	-3.4870065	-0.2272486	0.0000000

H	-3.6552715	0.3917830	0.8832478
H	-3.6552715	0.3917830	-0.8832478
H	-4.2294951	-1.0423756	0.0000000
C	-1.8141064	-1.4568070	-1.1989389
H	-1.9951417	-0.8302035	-2.0754557
H	-0.7605756	-1.7482636	-1.1949171
H	-2.4170191	-2.3739558	-1.3053499
C	-1.8141064	-1.4568070	1.1989389
H	-0.7605756	-1.7482636	1.1949171
H	-1.9951417	-0.8302035	2.0754557
H	-2.4170191	-2.3739558	1.3053499

*Complex **29** (**1** + $MeNO_2$ + BF_3)*

C	1.0715504	0.4105133	1.1745627
C	-0.0048188	1.2650531	1.2035637
C	-0.5403919	1.6739930	0.0000000
C	-0.0048188	1.2650531	-1.2035637
C	1.0715504	0.4105133	-1.1745627
H	1.5845051	0.0258127	2.0427886
H	-0.4251027	1.5997672	2.1406941
H	-0.4251027	1.5997672	-2.1406941
H	1.5845051	0.0258127	-2.0427886
N	-1.7214723	2.5529366	0.0000000
O	-2.1606072	2.8794646	-1.0771545
O	-2.1606072	2.8794646	1.0771545
N	1.5651095	-0.0069057	0.0000000
O	2.5644191	-0.8658912	0.0000000
F	4.8718776	-1.1264671	0.0000000
F	3.9728905	0.6617663	1.1394123
F	3.9728905	0.6617663	-1.1394123
B	3.9711764	-0.1158642	0.0000000
N	-2.2346963	-1.5588655	0.0000000

O	-1.0322845	-1.7041679	0.0000000
O	-2.8108913	-0.4950231	0.0000000
C	-3.0534789	-2.7963419	0.0000000
H	-2.7771035	-3.3617883	-0.8878088
H	-2.7771035	-3.3617883	0.8878088
H	-4.1019946	-2.5185805	0.0000000

*Complex **30** (**1** + $MeNO_2$ + BF_3)*

C	0.6344576	-0.2760816	1.1764667
C	-0.6087424	0.3108761	1.2048919
C	-1.2198149	0.5957330	0.0001227
C	-0.6087752	0.3109459	-1.2046410
C	0.6344312	-0.2759767	-1.1763360
H	1.2248229	-0.5279955	2.0445863
H	-1.0903087	0.5468825	2.1427680
H	-1.0904080	0.5469780	-2.1425074
H	1.2246110	-0.5277032	-2.0446263
N	-2.5535806	1.2188181	-0.0000324
O	-3.0498113	1.4469966	-1.0775774
O	-3.0502795	1.4472443	1.0770431
N	1.2132082	-0.5646612	0.0000275
O	2.3969554	-1.1381635	-0.0000269
F	4.6938180	-0.7581377	-0.0000996
F	3.3405020	0.7170266	1.1396997
F	3.3405182	0.7170935	-1.1397501
B	3.5592748	-0.0265988	-0.0000474
C	-4.0079128	-1.6042302	-0.0000130
O	-4.9829660	-2.1590463	0.0000514
